# Tissue histology on the correlation between fracture energy and elasticity

**DOI:** 10.1007/s11548-023-03026-6

**Published:** 2023-10-19

**Authors:** Kenzo Yamamoto, Kazuaki Hara, Etsuko Kobayashi, Akagi Yuki, Ichiro Sakuma

**Affiliations:** 1 Graduate School of Engineering, The University of Tokyo, Tokyo, Japan; 2 Graduate School of Engineering, Tokyo University of Agriculture and Technology, Tokyo, Japan

**Keywords:** Biological tissue damage, Biomechanics, Fracture energy, Elasticity, Histology

## Abstract

**Purpose:**

Preemptively estimating tissue damage is crucial for a safe surgical procedure. We previously investigated the possibility of estimating the fracture energies of biological tissues based on their elasticities. However, the reason behind the presence of these correlations is poorly understood. In this study, we investigate the effect of a tissue’s histology on the correlation between the fracture energy and elasticity. We hypothesize that two tissues with similar fibrous structure will show a similar correlation between the fracture energy and elasticity.

**Methods:**

Porcine duodenum were used for this study. Two tensile tests were performed for each porcine duodenum specimen to determine its elasticity and tearing energy. The correlation between fracture energy and elasticity was then investigated using the results from the mechanical tests. Furthermore, duodenum specimens were fixed in 10% formalin while under tension. Microscopic images were then taken to visualize the fibrous structure within the duodenum tissues under tension.

**Results:**

The results from the tensile test showed that the fracture energy had an isotropic positive and linear correlation with the elasticity to the negative 0.5th power (*R*^2^ = 0.89), which was also previously reported in small intestinal (jejunum) specimens. Furthermore, the tearing patterns of the duodenum were identical to the ones reported in the jejunum. Hematoxylin and eosin staining on tissues fixed under tension showed that the endomysium fibers are involved in providing resistance toward traction.

**Conclusion:**

Through mechanical tests, we showed that porcine duodenum tissues also have a correlation between its fracture energy and elasticity. We also discussed that the histological structure of a tissue is an important factor that dictates how the tearing energy of a tissue will correlate to the elasticity. We understood that since the tearing mechanism between the duodenum and jejunum was similar, the correlations between their fracture energies and elasticities were also similar.

## Purpose

Minimally invasive surgeries are procedures where the surgeon performs surgical maneuvers through small incisions on the patient. Long surgical instruments (such as forceps and energy devices) and the endoscope are passed through trocars, which serves as a gate between the environment inside and outside of the patient’s body. As the surgeon is deprived of direct contact with the patient’s biological tissues, minimally invasive surgery results in a significant lack of haptic feedback [1, 2] and a limited field of view [3] for the surgeon. This lack of haptic feedback and the limited field of view restricts the surgeon to perform surgical skills [4] such as the force control skill, which we define as the ability to estimate the maximal applicable force onto a tissue before inducing damage. This skill is important, as it prevents the surgeon from inducing unwanted damage onto healthy tissues during surgical maneuvers. Therefore, the development of a model to preemptively estimate tissue damage is of significant interest because it will allow the implementation of the force control skill into minimally invasive surgery.

A model that will be viable for the implementation of the force control skill into minimally invasive surgery must utilize information that are available during surgery. The implementation of force sensors into surgical forceps [5] and the reconstruction of tissue deformation [6] are examples of studies with the aim of quantifying the available information during surgery. Such intra-operative quantitative measurements will allow surgeons to determine physical properties, such as tissue elasticity. Therefore, the goal of this study is to preemptively estimate tissue damage using its elasticity as an input.

We previously investigated the possibility of estimating tissue damage based on tissue elasticity on porcine jejunum [7] and porcine aortic tissues [8, 9], in view of incorporating the force control skill into minimally invasive surgery. Through mechanical tests, we reported that a positive and linear correlation between tissue elasticity ($E$) and tearing energy (${T}_{\mathrm{o}}$) existed in small intestinal (jejunum) tissues as follows [7].

1 $$ T_{{\text{o}}} \propto E^{ - 0.5} $$

Furthermore, we also showed that a positive and linear correlation existed between $T_{{\text{o}}}$ and $E$ existed in porcine aortic tissues as follows [8, 9]:

2 $$ T_{{\text{o}}} \propto E $$

However, the reason behind the two above correlations has not been elucidated. As a first step toward understanding the previously reported results, we raised the following two questions, which will be assessed in this paper: Are there other tissues that exhibits the correlations shown in Eqs. (1) and (2)? What dictates whether the ${T}_{\mathrm{o}}$ of a tissue will correlate with either $E$ or ${E}^{-0.5}$? Our hypothesis is that other tissues will also show a correlation between ${T}_{\mathrm{o}}$ and $E$ or ${E}^{-0.5}$. Also, since biomechanical properties are mainly dictated by the quantity and structure of the fibrous proteins within the tissue, we hypothesize that the histological structure of a tissue is one of the factors that determines whether a given tissue will show a correlation similar to Eqs. (1) or (2).

## Approach

### Tissue selection

In this study, we decided to test on porcine duodenum tissues. The histological structure of the duodenum is similar to the jejunum. As both tissues are constituted of four distinctive layers: the serosa, muscularis, submucosa and mucosa. According to Egorov et al., the submucosa and the muscularis layers are the two layers that contribute to mechanical property [10]. The histological difference between the duodenum and the jejunum is the presence of Brunner’s glands in the submucosal layer in the duodenum. They consist of cells that are arranged as lobules and are often surrounded by reticular and elastic fibers [11]. Although the presence of Brunner’s glands, the overall histological structures of the duodenum and the jejunum are considered to be similar.

Since biomechanical properties are mainly dictated by the fibrous proteins within the tissue, we expect that a positive and linear correlation between ${T}_{o}$ and ${E}^{-0.5}$ (Eq. 1) would also be present in the duodenum, which would validate our hypotheses. Furthermore, since the histological structure are considered to be similar, we anticipate that the way how the tissue tears in the duodenum will resemble the one reported in the jejunum as well [7].

### Experimental design

**Fig. 1:**
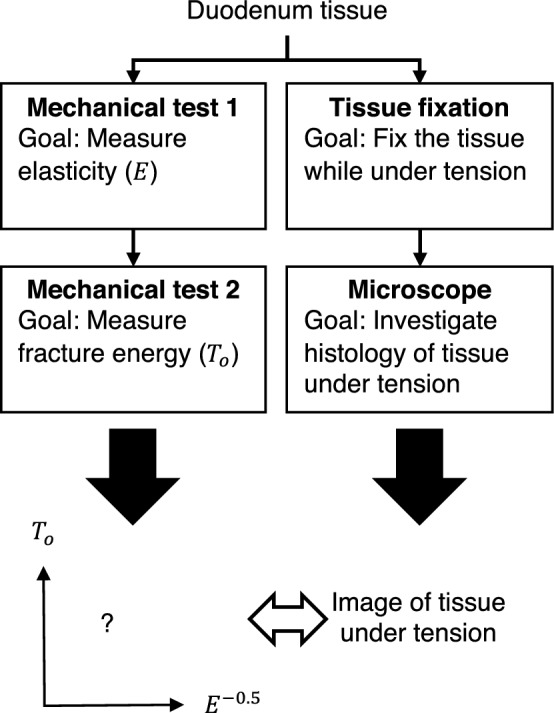
Overview of the experiments. Porcine duodenum samples underwent a succession of tensile tests to determine $E$ in Mechanical test 1 and ${T}_{\mathrm{o}}$ in Mechanical test 2. The ${T}_{\mathrm{o}}$ from each sample was then plotted against the $E$ determined from the same specimen. In parallel to the mechanical tests, trouser-shaped duodenum samples were fixed in 10% formalin to preserve the internal structure of the tissue while under tension. The samples were then microscopically observed for histologic analysis. This was done in order to gain a histologic understanding of the biomechanical results obtained

The mechanical property of a biological tissue is heterogeneous [12]. Meaning that the property between two different samples harvested from the same tissue will differ. Measuring the corresponding ${T}_{\mathrm{o}}$ for an $E$ using two different samples is therefore an unviable option. It is therefore imperative that ${T}_{\mathrm{o}}$ and $E$ are both measured using the same tissue samples. To this effect, two mechanical tests were performed per sample. The first tensile test was done to determine $E$ and the second test was conducted to calculate ${T}_{\mathrm{o}}$. Strip-shaped and trouser-shaped specimens were used for the first test and second tensile tests, respectively. It is important to note that, in theory, performing two tensile tests on the same specimen should not have an influence of the mechanical properties of the biological tissue if the elongation of the first tensile test is kept under the yield points of the specimens tested [13]. Furthermore, microscopic observations on the duodenum tissues fixed while under tension were performed as a first step toward gaining a histologic understanding of the biomechanical results. The overview of the experimental design, as well as the goal of each step, is shown in Fig. 1.

## Methods

### Sample preparation

Porcine duodenum tissues were provided by Tokyo Shibaura Zouki Ltd., Tokyo, Japan in cold containers 12 h post-mortem. Sample preparation for the tensile tests was conducted immediately upon arrival, and the tests were performed within 12 h upon arrival.

After cutting open the duodenum, 12 strip-shaped specimens each from the 0$^\circ$, 45$^\circ$, and 90$^\circ$ angles relative to the longitudinal axis were harvested. 50 mm × 8 mm strip-shaped specimens were cut out using a stencil and surgical tools. The thickness of each specimen was recorded, and the test pieces were preserved in ice-cold PBS prior to the first tensile test to determine $E$. Following the first tensile test, an incision was made on the width-side of the strip-shape specimens using a sharp scalpel and a custom-made stencil to create the trouser-shaped specimens. The trouser-shaped specimens were preserved in ice-cold PBS before moving onto the second tensile test.

### Mechanical tests

All mechanical tests were performed using a tensile tester (EZTest/CE-346-51990, Shimadzu Corporation, Kyoto, Japan) equipped with a 500 N load cell (SM-500N-168, Shimadzu Corporation, Kyoto, Japan). The tissues were fixed onto the testing platform using the appropriate holders, and sandpaper was set between the tissue and the clamps to prevent slippage. The strip-shaped specimens were clamped at both extremities of the sample, and the trouser-shaped specimens were held by their legs. An initial tension of 0.01 N was set on the biological tissue prior to starting the tensile test, to begin every test on the same experimental basis. Tensile tests were performed under a constant stroke rate of 50 mm min^−1^ at room temperature.

### Mechanical characteristic calculation

The results from the mechanical tests resulted in a load-extension curve. The load-extension curve from the first tensile test was converted into a stress–strain curve. The stress was obtained by dividing the load by the cross-sectional area of the strip-shaped specimen (width $\times$ specimen thickness). The strain was calculated by dividing the extension by the initial length of the strip-shaped specimen. $E$ was calculated from the linear region of the low-strain region of the stress–strain curve resulting from the first tensile test. The linear regression function in Microsoft Excel (version 16.66.1, Microsoft co. ltd) was used to plot the trendline of the linear region. The slope of the trendline (with an ${R}^{2}>0.9$) was used as $E$.

The load-extension curve of the second tensile test was used to calculate ${T}_{\mathrm{o}}$. ${T}_{\mathrm{o}}$ is defined as the energy necessary to produce a new surface resulting from the propagation of a tear. The energy necessary to induce a tear is the product between the force during the tear propagation ($F$) and the tearing distance ($\Delta x$). $F$ was calculated as the local minimum average during the tear propagation. This method was taken to minimize the effect from the stick–slip behavior, as suggested by Sakai et al. [14]. The new surface resulting from the tear propagation was calculated as the product between the thickness of the tissue ($h$) and $\Delta x$. In the context of this study, the distance at which $F$ is being applied ($\Delta x$ in the numerator) was assumed to be equal to the tearing distance ($\Delta x$ in the denominator). The equation for ${T}_{\mathrm{o}}$ is as follows:

3 $$ T_{o} = \frac{F \times \Delta x}{{h \times \Delta x}} $$

### Microscopic observation

**Fig. 2:**
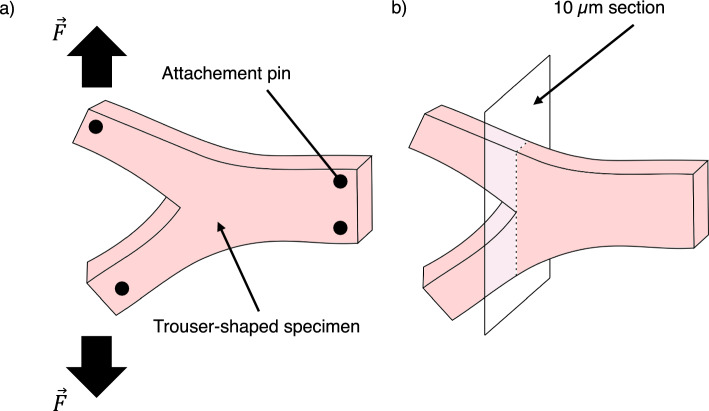
Tissue preparation process for microscopic observation. **a** The tissue is first tensioned and fixed in 10% formalin using attachment pins. **b** 10 µm sections near the fracture propagation tip were prepared for tissue observation

Microscopic observations were performed to investigate the fibrous structure of the duodenum when the tissue is under tension. The trouser-shaped duodenum tissues were pulled apart and fixed in 10% formalin (Fujifilm Wako Pure Chemical Corporation, Osaka, Japan) using pins on a silicone mat (Fig. 2a). The tissues were fixed for 16 h at room temperature. Tissues were then embedded in embedding medium (O.C.T. Compound, Sakura Finetek Japan, Tokyo, Japan) and cryosectioned at $10\; \upmu \text{m}$ thickness using a cryostat (CM3050S, Leica Biosystems, Wetzlar, Germany) (Fig. 2b). The sections were placed on microscopic slides (APS-03 15, Matsunami Glass Ind., Ltd., Osaka, Japan) for observation. Sections were stained with hematoxylin and eosin (ScyTek Laboratories, Utah, USA) following the protocol suggested by the supplier. The stained sections were observed using an optical microscope (Eclipse Si, Nikon Corporation, Yokohama, Kanagawa, Japan) and photographs of the microscopic images were taken using a C-mount camera mounted onto the microscope (Digital Sight 1000, Nikon Corporation, Yokohama, Kanagawa, Japan).

### Image analysis

All images (microscopic images and photographs) were analyzed using Fiji [15]. Fiber orientation in microscopic images was determined by transforming the data into a binary image. The ROI was a square (500 pixels $\times$ 500 pixels) set in a way where both muscularis layers were selected. The directionality plugin, which utilizes the method presented by Liu [16], was utilized to determine the fiber orientation distribution. Furthermore, tearing angles were obtained by measuring the angle of manually selected segments along the incision made to create the trouser-shaped specimens and the tearing line in specimen photographs after the second tensile test.

### Statistical analysis

The linear regression analyses between the measured values of ${T}_{\mathrm{o}}$ and ${E}^{-0.5}$ in porcine duodenum specimens were performed using Microsoft Excel (version 16.66.1, Microsoft co. ltd). Analysis of covariance (ANCOVA) was performed to compare the slopes of the trendlines resulting from the linear regression using MATLAB r2020b (The MathWorks, 2020). One-way analysis of variance (ANOVA) and post-hoc analyses (Tukey–Kramer test) were also performed using MATLAB r2020b (The MathWorks, 2020) to assess the specimens’ tearing angles after the second tensile test.

## Results

### Tearing patterns

**Fig. 3:**
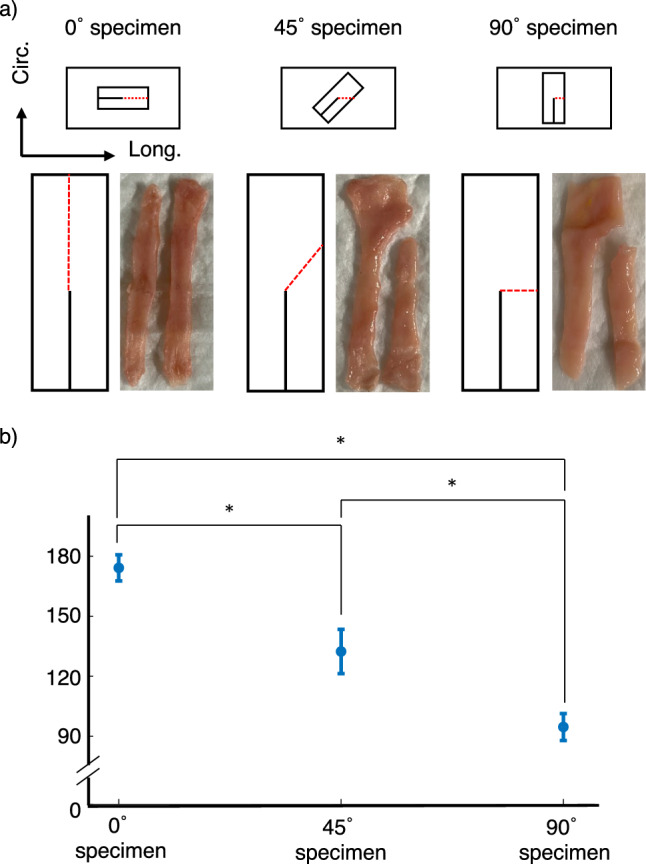
**a** Photographs of the tearing patterns observed in porcine duodenum tissues after the second tensile test on trouser-shaped specimens. The red dotted lines represent the propagation of the tear. **b** Tearing angles relative to the incision made to create the trouser-shaped specimens. The Tukey–Kramer test showed that the tearing angles significantly differed from one another (*p* < 0.05)

Specific tearing patterns associated to the harvesting angles were observed among the trouser-shaped specimens after the second tensile test, as shown in Fig. 3a. The tear propagated along, at an angle, and perpendicularly in samples harvested in the 0$^\circ$, 45$^\circ$ and 90$^\circ$ orientation relative to the duodenum’s longitudinal axis, respectively. The average tearing angles relative to the incision made to create the trouser-shaped specimens are shown in Fig. 3b. The tearing angle for the 0$^\circ$ specimens, 45$^\circ$ specimens and 90$^\circ$ specimens were $174.2\pm 6.5^\circ$ ($n=5$), $132.4\pm 11.1^\circ$ ($n=5$) and $94.6\pm 6.7^\circ$ ($n=5$), respectively. The one-way ANOVA test resulted in a p-value of $p=1.64\times {10}^{-8}$, when comparing the tearing angles from all harvesting angles. The post-hoc Tukey–Kramer test outputted a p-value of $1.18\times {10}^{-5}$ when comparing the tearing angles from the specimens harvested in the 0$^\circ$ and 45$^\circ$ angles, $1.30\times {10}^{-8}$ after comparing the 0$^\circ$ and 90$^\circ$ angle dataset, and $3.31\times {10}^{-5}$ between the 45$^\circ$ and 90$^\circ$ angles.

### Elasticity and fracture energy in porcine duodenum

**Fig. 4:**
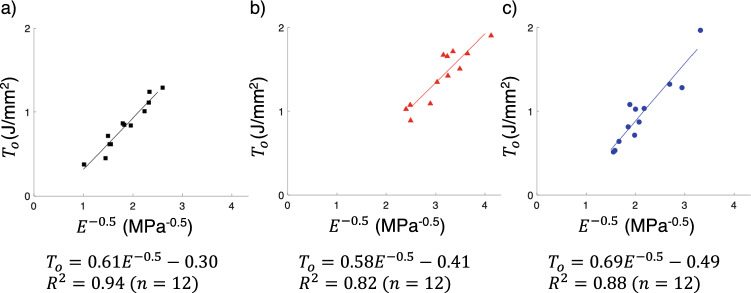
${T}_{\mathrm{o}}$ as a function of ${E}^{-0.5}$ based on the results from the mechanical tests on porcine duodenum tissues harvested in the **a** 0° orientation, **b** 45° orientation and **c** 90° orientation, relative to the longitudinal axis

The results for ${E}^{-0.5}$ and ${T}_{o}$ obtained using porcine duodenum tissues are shown in Fig. 4a–c. The black squares, red triangles and blue dots represent the data acquired from the specimens harvested in the 0$^\circ$ ($n=12$), 45$^\circ$ ($n=12$) and 90$^\circ$ ($n=12$) orientation relative to the longitudinal axis of the small intestine, respectively. The R^2^ values from the linear regression on the 0$^\circ$, 45$^\circ$ and 90$^\circ$ datasets were $0.93$, $0.82$ and $0.88$, respectively. The trend line on the 0$^\circ$ dataset was ${T}_{\mathrm{o}}=0.61{E}^{-0.5}-0.30$, while the ones from the 45$^\circ$ and 90$^\circ$ datasets were ${T}_{o}=0.58 {E}^{-0.5}-0.41$ and ${T}_{o}=0.69 {E}^{-0.5}-0.49$, respectively. The results from the ANCOVA test when comparing the slopes between the trendlines from the harvesting angle-dependent datasets resulted in a p-value of $0.86$. The *R*^2^ value for the entire dataset was 0.89 for a sample size of 36, and the regression line equation was ${T}_{\mathrm{o}}=0.53{ E}^{-0.5}-0.18$.

### Microscopic observations and fiber orientation

**Fig. 5:**
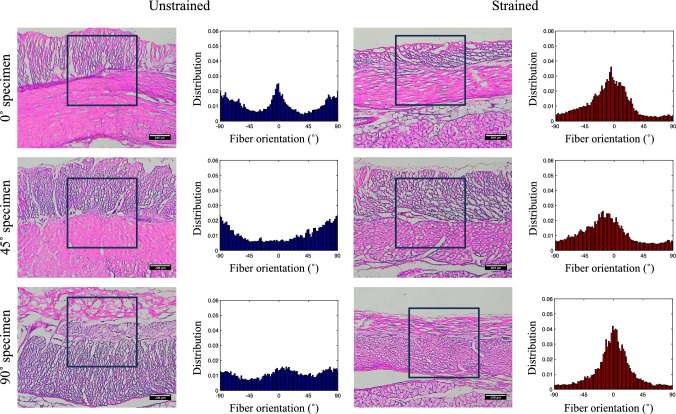
Microscopic observations of the duodenum in the unstrained (left) and strained (right) state and fiber orientation distribution (unstrained in blue and strained in red) of the specimen harvested from the 0° angle orientation (top row), 45° orientation (middle row), and 90° orientation (bottom row). The black squares on the microscopic images represent the ROI for the fiber orientation analysis

The microscopic images of the duodenum specimens in the unstrained and strained state, as well as the distribution of the fiber orientation obtained from the ROI (black squares), are compiled in Fig. 5. The results from the specimens harvested in the 0$^\circ$, 45$^\circ$ and 90$^\circ$ orientation relative to the duodenum’s longitudinal axis are shown in the top, middle and bottom rows, respectively. A peak in fiber orientation can be observed at − 90.00°, 90.00°, and − 1.01° in the unstrained specimen harvested in the 0° orientation. Whereas the strained specimen from the same harvesting orientation showed a peak at − 5.06°. For the specimen harvested in the 45° orientation, peaks at − 90.00° and 90.00° can be seen in the unstrained state, and a peak at − 11.12° was observed in the specimen in the strained state. As for the specimen harvested in the 90$^\circ$ orientation, no preferential fiber orientation was observed in the unstrained state, and a peak at − 1.01° was present in the strained state.

## Discussion

### Relationship between elasticity and fracture energy in porcine duodenum

The first research question we aimed to answer is whether or not the correlations between ${T}_{o}$ and $E$ shown in Eqs. (1) and (2) would exist in other biological tissues. The series of mechanical tests on porcine duodenum tissues resulted in a linear and positive correlation between ${T}_{\mathrm{o}}$ and ${E}^{-0.5}$ as shown in Fig. 4a–c. From these results, we can say that our first hypothesis stating that either correlation shown in Eqs. (1) or (2) can be obtained using a tissue different from the aorta and the jejunum is validated. Furthermore, the ANCOVA test suggested that there were no statistically significant differences among the slopes of the trend lines obtained from the linear regression performed on harvesting angle-dependent datasets. Therefore, the correlation between ${T}_{\mathrm{o}}$ and ${E}^{-0.5}$ in the duodenum presented in this study is isotropic.

The linear regression using the entire dataset from the mechanical tests on duodenum tissues resulted in the following trendline: ${T}_{\mathrm{o}}=0.53 {E}^{-0.5}-0.18$. The trendline reported using the jejunum was ${T}_{\mathrm{o}}=0.72 {E}^{-0.5}-0.30$ [7]. The slopes and ${T}_{\mathrm{o}}$-intercepts obtained for both tissues are of the same order. These results suggest that the major tissue constituent that plays a role in dictating $E$ and ${T}_{\mathrm{o}}$ in both the duodenum and jejunum share similar physical properties.

One difference between the results from the duodenum and jejunum is the fact that the slope of the trend line from the results obtained in the samples harvested in the 45$^\circ$ angle in the jejunum was significantly different from the ones in the 0$^\circ$ and 90$^\circ$ orientations [7]. The presence of anisotropy in the jejunum compared to the isotropic correlation found in the duodenum suggests that although the protein fibers dictating the values of $E$ and ${T}_{\mathrm{o}}$ in both tissues are similar, their configuration in the fibrous network in which they consist of might differ. An extensive histologic assessment of the fibrous network in the jejunum and duodenum must be conducted in order to elucidate this difference between the results found in both tissues.

### Tearing patterns in porcine duodenum

When observing the tearing patterns of the trouser-shaped duodenum specimens after the second tensile test, the measured tearing angles for each harvesting angle-dependent dataset were different from one another (Fig. 3a). In fact, the one-way ANOVA test suggested that the tearing patterns were statistically different when comparing the tearing angles from all harvesting angles with a *p* value of $1.64\times {10}^{-8}$. Furthermore, the post-hoc Tukey–Kramer test suggested that a significant difference existed between the tearing angles from the specimens harvested in the 0° and 45° angles ($p=1.18\times {10}^{-5}$), 0° and 90° angles ($p=1.30\times {10}^{-8}$), and 45$^\circ$ and 90° angles ($p=3.31\times {10}^{-5}$), as shown in Fig. 3b. Although the correlation between ${T}_{o}$ and ${E}^{-0.5}$, in the duodenum, did not show any anisotropy, the direction of the tear in the trouser-shaped specimens did. These results suggest that fracture propagates through the duodenum tissues by stretching and breaking an isotropic network of fibrous protein located between stiffer and stronger fibers which are organized in an anisotropic way within the tissue. These results were also observed in the jejunum, with identical tearing patterns [7]. These findings suggest that the tearing mechanism in the duodenum and jejunum are identical. Meaning that these tissues tear by stretching a network of fibrous proteins located between fibers that are oriented along the tear propagation.

When observing the tearing patterns in the duodenum (Fig. 3a) and the jejunum [7], the tear propagates in the same direction as the longitudinal axis of these tissues. Collagen fibers in the submucosal layer in both tissues are mostly longitudinally oriented [17]. Collagen fibers are considered to be stiff fibers and do not break easily. It would thus be reasonable to state that the duodenum and jejunum tear down the collagen fibers, by breaking whatever fibrous network in between. In addition, Egorov et al. reported that the submucosa and the muscularis layers are the two layers that contribute to the mechanical properties of the gastrointestinal tract [10]. Since collagen fibers do not break during tissue tearing, the network of fibers in the muscularis layers must be the main contributor toward the resistance toward tissue tearing, and therefore ${T}_{o}$. The muscularis layers consist of an inner layer of smooth muscle cells that are circumferentially oriented, and an outer layer where the smooth muscle cells are longitudinally oriented. These smooth muscle cells are connected by a fibrous protein called endomysium fibers.

The tearing patterns observed after the second tensile test on trouser-shaped specimens, as well as the histologic understanding of the tissues in question suggests that the duodenum and the jejunum tear by breaking the endomysium fibers. Since both tissues share the same tearing mechanism and showed a similar overall correlation between ${T}_{\mathrm{o}}$ and ${E}^{-0.5}$, it can be stated that the configuration of the network of fibers that undergoes damage during tissue tearing plays a role in dictating whether the ${T}_{\mathrm{o}}$ of a tissue will correlate with $E$ or ${E}^{-0.5}$. It is therefore important to investigate the implication of the endomysium network in the mechanical property of these tissues.

### Histologic study of the tissues under tension

Microscopic images of porcine duodenum tissues were taken to investigate the behavior of the endomysium fiber network when the tissue is under tension. To interpret the results shown in Fig. 5, it is important to note that the tensile direction in these microscopic images was in the 0.00° direction. In the unstrained state, a significant proportion of the fibers within the ROI is oriented in the vertical direction (± 90.00°). However, in the strained state, most of the fibers within the ROI tend toward 0.00$^\circ ,$ i.e., the tensile direction.

From these results, it can be suggested that when duodenum tissues undergo tensile deformation, the endomysium fibers in the muscularis layers reorient themselves along the tensile direction. The resistance toward the tensile deformation comes from the mechanical property of individual fibers and the bulk property resulting from the network formed by these fibers. This point is further supported by the fact that a single peak near the 0.00$^\circ$ orientation resulted from the analysis in all harvesting directions, since the initial orientation of smooth muscle cells, and hence the endomysium fibers, would differ based on the harvesting angle.

As mentioned earlier, the muscularis layers and the submucosal layers are the two layers that contribute to the mechanical properties of small intestinal tissues [10]. Furthermore, it was experimentally demonstrated that the tearing directions differed based on the harvesting angle, due to the collagen fiber orientation in the submucosal layer. The present histologic study on the duodenum tissues under tension suggests that the endomysium fibers reorient themselves in the tensile direction to provide resistance toward traction. These results further support the proposed tearing mechanism of duodenum tissues, where the tissue tears by breaking the endomysium fibers in the muscularis layers and the direction of the tear is guided by the collagen fibers in the submucosal layer.

### Limitations and future direction

There are multiple limitations in this study. The following limitations must be addressed in the future in order to successfully incorporate the force control skill into robotic-assisted surgery.

The first limitation is the fact that the tearing distance ($\Delta x$ in the denominator in Eq. 1) was assumed to be equal to the distance at which the traction force $F$ is being applied ($\Delta x$ in the numerator in Eq. 1). This assumption would only be valid for the 90$^\circ$ specimen because the tear in these samples tear down the incision that was made to create the trouser-shaped specimens (Fig. 3a). Since our results showed that the tearing direction significantly varies depending on the harvesting angle of the specimen (Fig. 3b), this assumption would be false for the specimens harvested in the 0$^\circ$ and 45$^\circ$ orientations. To circumvent this issue, video tracking of the specimens should be conducted in future works to independently measure the tearing distance during the tensile tests.

In addition, there is a limitation regarding the fact that the same tissue sample underwent two successive tensile tests. Although the extent at which the tissue was pulled during the first tensile test was low, the possibility of the internal structure of the tissue undergoing plastic deformation is not null. It is therefore desirable to obtain the values of ${T}_{\mathrm{o}}$ and $E$ of a sample using a single tensile test. However, obtaining the $E$ of a trouser-shaped specimen is difficult because the force applied onto the specimen is not uniformly distributed throughout the sample. One possible approach to circumvent the issue of performing multiple mechanical tests on the same sample would be to measure the $E$ of a tissue using non-contact methods [18].

Furthermore, only one type of deformation was tested on the trouser-shaped specimens. During surgery, multiple forces are applied onto the tissue, such as traction force, shear force and compressive force. In order to incorporate a force control skill into robot-assisted surgery, the influence of the type of force on the correlation between ${T}_{\mathrm{o}}$ and $E$ must be investigated. In addition, biological tissues have viscoelastic properties. Meaning that the speed at which the force is being applied would significantly influence the mechanical response. Considering the fact that tissue handling speed is a continuously varying parameter during surgery, the effect of deformation speed should also be addressed during the development of this model.

Regarding the proposed tearing mechanism in the duodenum, one major assumption in this study is that the muscularis layers are the main mechanical contributors in the resistance toward tissue tearing. Meaning that the collagen fibers in the submucosal layer were considered to be independently arranged from one another. The possibility, that the resistance provided by breaking the connection between the collagen fibers in the submucosal layer is also included in the calculation of ${T}_{\mathrm{o}}$, cannot be neglected. Therefore, to better understand the correlation between ${T}_{\mathrm{o}}$ and ${E}^{-0.5}$, it is important to answer the following question: What histological constituents are involved during the calculation of ${T}_{\mathrm{o}}$ and $E$? This question can be answered by obtaining a temporal sequence of microscopic images of the tissue’s histology while performing a mechanical test. In this study, the tissues were fixed under tension in 10% formalin. Therefore, microscopic observation of tissues without the need of tissue fixation and staining must be explored in the future.

## Conclusion

This study aimed to answer two questions. Does a correlation between ${T}_{\mathrm{o}}$ and $E$ exist in tissues other than the aorta and jejunum? If it does, what dictates whether the ${T}_{\mathrm{o}}$ of a tissue will correlate with either $E$ or ${E}^{-0.5}$? Mechanical tests were performed on porcine duodenum tissues and the results showed a positive and linear correlation between ${T}_{\mathrm{o}}$ and ${E}^{-0.5}$, as in the jejunum. Histological structure similarities between both tissues were also discussed. From this, we conclude that a correlation between ${T}_{\mathrm{o}}$ and $E$ does exist in tissues other than the aorta and jejunum, and that the histologic structure of the tissue undergoing damage is one of the main parameters that dictates whether the ${T}_{o}$ of a tissue will correlate more toward $E$ or ${E}^{-0.5}$. This paper also highlights the importance of observing the histologic structure of a tissue while performing mechanical tests. As such information can lead to valuable evidence toward understanding the mechanism of tissue deformation and damage. Thus, consequently leading toward the implementation of biomechanical tissue models in view of increasing the overall quality of minimally invasive surgery.
